# Repeated evolution of durophagy during ichthyosaur radiation after mass extinction indicated by hidden dentition

**DOI:** 10.1038/s41598-020-64854-z

**Published:** 2020-05-08

**Authors:** Jian-dong Huang, Ryosuke Motani, Da-yong Jiang, Xin-xin Ren, Andrea Tintori, Olivier Rieppel, Min Zhou, Yuan-chao Hu, Rong Zhang

**Affiliations:** 1 Department of Research, Anhui Geological Museum, Jiahe Road 999, Hefei, Anhui 230031 China; 2 Department of Earth and Planetary Sciences, University of California, One Shields Avenue, 95616 Davis, California USA; 3 Department of Geology and Geological Museum, Peking University, Yiheyuan Street. 5, Beijing, 100871 P.R. China; 4 State Key Laboratory of Palaeobiology and Stratigraphy (Nanjing Institute of Geology and Palaeontology), Chinese Academy of Science, Nanjing, 210008 P. R. China; 5 Dipartimento di Scienze della Terra, Università degli Studi di Milano, Via Mangiagalli 34-20133, Milano, Italy; 6 Center of Integrative Research, The Field Museum, Chicago, IL 60605-2496 USA

**Keywords:** Palaeontology, Palaeoecology, Palaeoecology

## Abstract

Marine tetrapods quickly diversified and were established as marine top predators after the end-Permian Mass extinction (EPME). Ichthyosaurs were the forerunner of this rapid radiation but the main drivers of the diversification are poorly understood. *Cartorhynchus lenticarpus* is a basal ichthyosauriform with the least degree of aquatic adaptation, holding a key to identifying such a driver. The unique specimen appeared edentulous based on what was exposed but a CT scanning revealed that the species indeed had rounded teeth that are nearly perpendicular to the jaw rami, and thus completely concealed in lateral view. There are three dental rows per jaw ramus, and the root lacks infoldings of the dentine typical of ichthyopterygians. The well-developed and worn molariform dentition with three tooth rows supports the previous inference that the specimen is not of a juvenile. The premaxilla and the corresponding part of the dentary are edentulous. Molariform dentition evolved three to five times independently within Ichthyosauriformes in the Early and Middle Triassic. Convergent exploitation of hard-shelled invertebrates by different subclades of ichthyosauriforms likely fueled the rapid taxonomic diversification of the group after EPME.

## Introduction

Many components of the modern ecosystem emerged in the Triassic, after the EPME. One of them is marine tetrapods, air-breathing vertebrates that invaded the sea from land, such as marine mammals and reptiles^1^. Marine colonization by tetrapods occurred at least 69 times in the past, 27 of which were in the Mesozoic^2^. A high concentration of such colonization events is found soon after the EPME in the Early to Middle Triassic, when multiple lineages of marine tetrapods entered marine environments and radiated quickly to achieve high taxonomic and ecological diversity^3^. Some of these lineages gave rise to the iconic marine reptiles of the Jurassic and Cretaceous, such as ichthyosaurs and plesiosaurs, that occupied niches similar to those of cetaceans and pinnipeds in the modern sea^4^. However, it is still unclear what may have fueled this rapid early diversification.

**Figure 1:**
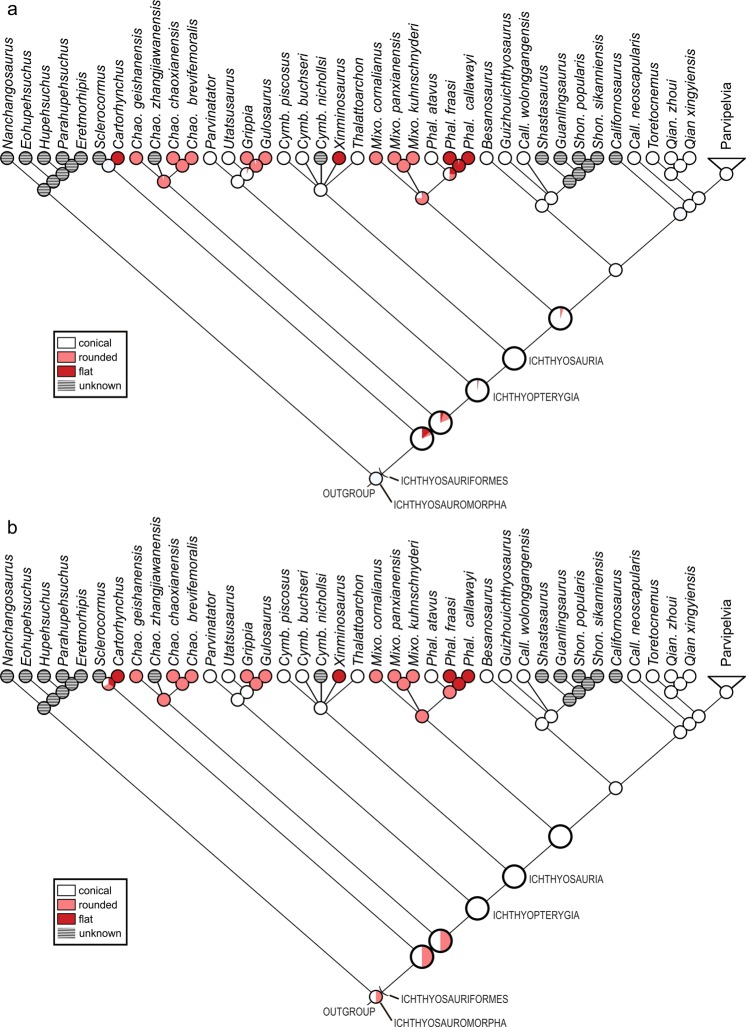
Ancestral state reconstruction of the posterior tooth crown shape. (**a**) Likelihood reconstruction. (**b**) Parsimony reconstruction. See Methods.

Ichthyosaurs are a group of marine reptiles noted for the evolution of fish-shaped body profiles^5^. Typical fish-shaped ichthyosaurs form the clade Parvipelvia, a subclade within Ichthyosauria, which in turn is a part of Ichthyopterygia^6^ (Fig. 1). The sister group of ichthyopterygians has been ambiguous but it is now likely that Nasorostra, a recently discovered clade of marine reptiles, is sister to Ichthyopterygia, together belonging to a lineage called Ichthyosauriformes^7^. *Cartorhynchus lenticarpus* was the first nasorostran to be discovered, followed by *Sclerocormus parviceps*^7,8^. Nasorostra are the only ichthyosauriforms to have a combination of features that are expected in the earliest members of the clade soon after marine invasion, such as the abbreviated snout, short body trunk, and pachyostotic ribs. However, Nasorostra have been known only for four years based on two specimens, so our knowledge of the group is still limited. Yet, they are expected to provide information on the emergence of Ichthyosauriformes because of their basal position within the clade. Additional information on this clade is clearly needed.

One of the controversies about the basal ecomorphology of ichthyosaurs, dating back to the previous century, concerns the dentition. It was once believed that rounded posterior teeth are plesiomorphic in this lineage, largely based on the condition found in *Grippia longirostris*^9^ that was then believed to be the most basal ichthyosaur, but later discoveries revealed that some other basal members of the clade had conical teeth while *G. longirostris* was not necessarily the most basal ichthyopterygian^6,10,11^. Of the four-known basal ichthyopterygians from the Early Triassic, two have rounded posterior teeth and the other two have conical teeth. Ichthyopterygia in this context accords with the first phylogenetic definition explicitly given to the clade^6^, i.e., a clade containing all descendants of the last common ancestor of *Utatsusaurus*, *Grippia*, and *Ichthyosaurus* (Fig. 1). Dental morphology usually reflects diet and other ecological factors and may provide important information on what fueled the early radiation of marine tetrapods in the Early and Middle Triassic. It is therefore important to understand the evolution of dentition in early marine reptiles.

*Cartorhynchus lenticarpus* is known only from a single specimen exposed from the right-dorsal side^8^. The mouth is closed, with up to one millimeter of a gap between the upper and lower jaws depending on the location. No dentition was seen through the gap, so it was initially considered edentulous^8^. An attempt to remove further matrix through this gap revealed that there was a tooth-like structure, which later turned out to be an isolated tooth that was not attached to the jaws. The discovery prompted an examination of the hidden left side of the specimen to test the presence of a dentition. However, the holotype and only known specimen is delicate, so mechanical preparation from the other side would involve risk of damage. We therefore decided to CT scan this rare specimen. The purpose of the present paper is to report the anatomical features revealed by CT scanning, including a unique dentition, and discuss the evolution of tooth morphology and diet in basal ichthyosauriforms and its bearing on the rapid diversification of the clade after the end-Permian mass extinction.

## Results

### Preservation

**Figure 2:**
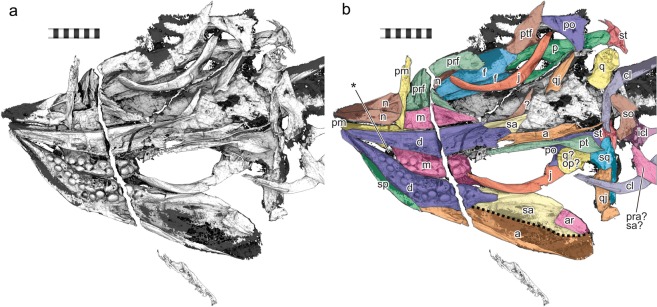
3D rendering of the hidden side of the holotype of *Cartorhynchus lenticarpus* (AGB 6257), revealing the dentition. (**a**) Volume rendering using 2d transfer function on the CT slices. (**b**) Surface mesh rendering based on Sequential Isosurface Trimming (see Methods). (**c**) Same as b with some cranial bones identified. Abbreviations: **a** angular; **ar**, articular; **cl**, clavicle; **co**, coronoid; **d**, dentary; **f**, frontal; **icl**, interclavicle; **j**, jugal; **m**, maxilla; **n**, nasal; **p**, parietal; **pm**, premaxilla; **po**, postorbital; **pra**, prearticular; **prf**, prefrontal; **ptf**, postfrontal; **q**, quadrate; **qj**, quadratojugal; **sa**, surangular; **so**, supraoccipital; **sp**, splenial; **sq**, squamosal; **st**, supratemporal. Scale bar in 1 cm.

The specimen has severely been compacted to about 7 mm of total thickness. As a result, boundaries between bones are often unclear in CT images when a bone is preserved on top of another. Unlike the right side of the skull that is articulated, the left side, which was hidden previously and revealed by CT scanning for the first time, is disarticulated, although many bones are still located close to the original positions. Thus, it is likely that the specimen was deposited with the right side facing down and embedded in sediment, while the left side was exposed for longer and became disarticulated. Some posterior cranial bones are missing, including large parts of the left angular and surangular and most of the occiput. One possible mandibular element, tentatively identified as a possible prearticular in Fig. 2b, is found postcranially, overlapping the right clavicle, suggesting disturbance of the left-posterior part of the skull before complete burial.

### Tooth orientation and ‘occlusion’

**Figure 3:**
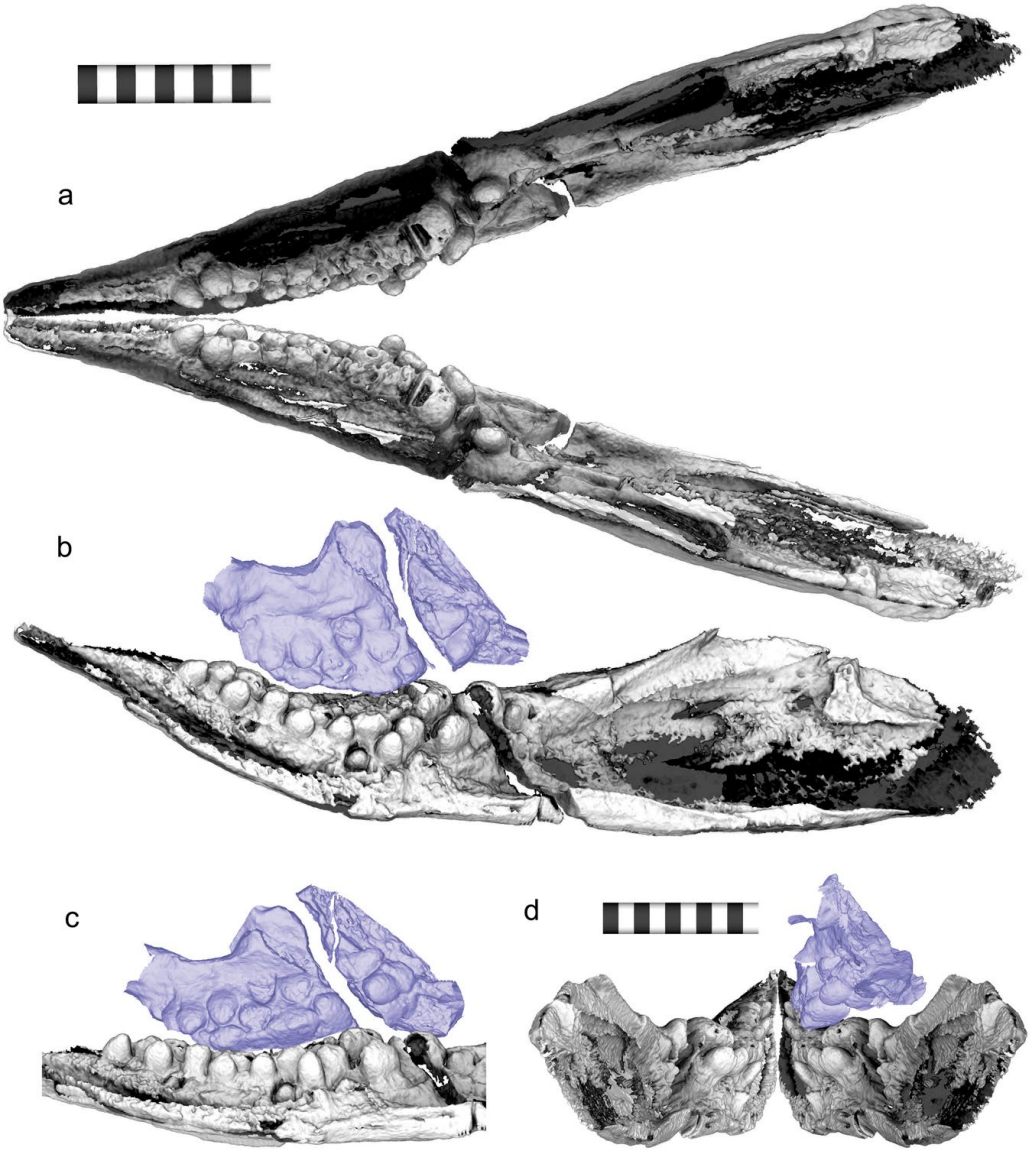
3D Reconstruction of mandibular posture. (**a**) Dorsal view of the reconstructed mandible. (**b**) Medial view of the reconstructed mandible and maxilla. (**c**) Medio-ventral view of the dentary and maxillary ‘occlusion’. (**d**) Same from posterior view. Maxilla is colored in purple. Scale bar is 1 cm.

The most unusual feature of the dentition is the orientation of the teeth. Unlike in most vertebrates, many teeth are perpendicular to the outer walls of the respective jaw rami, especially posteriorly (Fig. 3), and therefore completely concealed when viewing the ramus perpendicularly from its outer side. The tooth orientation in natural posture is more horizontal than vertical (Fig. 3), when it is nearly vertical in most reptiles. The outer walls of the jaw rami are preserved parallel to the bedding plane, with the teeth approximately vertical to the plane, i.e., compactional bias is unlikely to alter the orientation of the teeth (see Discussion).

**Figure 4:**
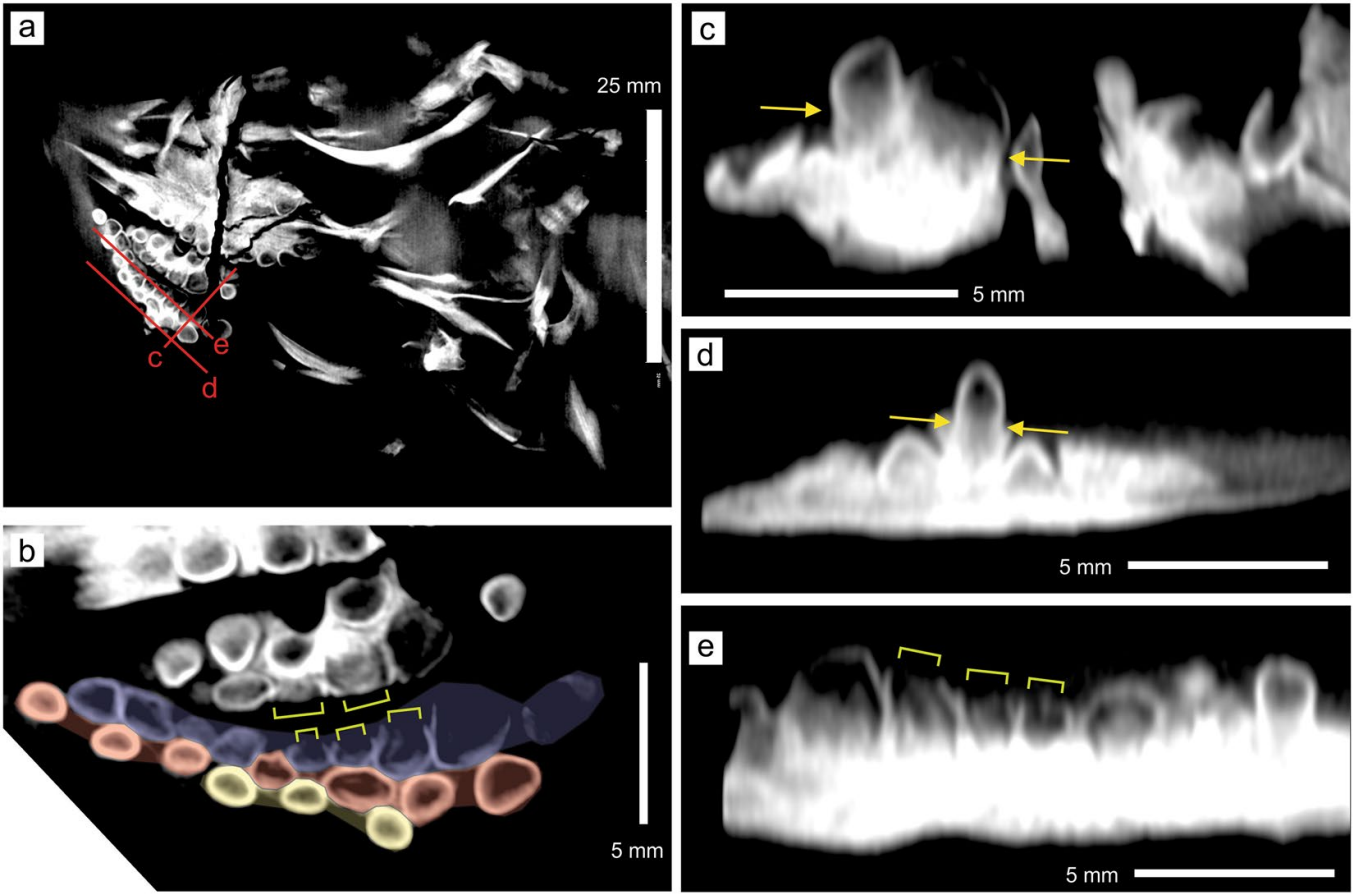
Sectional images of the 3D volume based on the CT images. (**a**) Section through the head region approximately parallel to the bedding plane. (**b**) A close-up view of the right dentary (below) and maxillary (middle to above) dentition in a, with a part of the left dentary teeth along the top margin. Dentary tooth rows are colored, with the labial row in blue, middle row in red, and lingual row in yellow. (**c**) A cross-section through the red line labeled c in a, nearly parallel to the longitudinal direction of the dentary teeth. (**d**) A cross-section through the red line labeled d in a, nearly parallel to the longitudinal direction of the dentary teeth. (**e**) A cross-section through the red line labeled e in a, nearly parallel to the longitudinal direction of the dentary teeth. Yellow allows point to the constriction between the crown and root. Yellow square brackets indicate the parts of tooth crowns without the enamel due to tooth wear.

The most-likely inclination of the mandibular rami is presented in Fig. 3 (see Methods). Because of the peculiar tooth orientation, the maxillary and dentary teeth do not contact each other tip to tip, and instead, ‘occlusion’ occurs between the side walls of the tooth crowns of the maxilla and dentary (see Discussion). This strange ‘occlusion’ is also evidenced by the tooth wear. Both maxillary and dentary teeth have been worn from their sides facing the corresponding dentition, and the worn maxillary dentition seems to fit into a shallow basin of worn dentary teeth (Fig. 3b–d). The worn teeth lack the thin wall of dense material on the occlusal side, which had most likely been worn through ‘occlusion’ (yellow brackets in Fig. 4b,e). The tooth wear is not an artifact of preservational compaction because the teeth outside of the occlusal area are not worn. The upper and lower teeth do not abut against each other in their preserved postures, so the wear was formed before the burial.

### Tooth morphology and arrangements

A total of 21 teeth are recognized on the right dentary, forming three rows that are approximately parallel to the jaw margin. There are 10, 7, and 4 teeth, respectively, in the labial, middle, and lingual rows (Fig. 4b; note that two immature teeth are not showing in this cross section, namely the most posterior tooth of the labial row and the second to the last tooth of the lingual row). The left dentary has 10, 7, and 3 teeth in the labial, middle, and lingual rows, respectively, but the count is less accurate given that they are not exposed on either side of the specimen. The antepenultimate tooth of the labial row of the right dentary is the largest, with the maximum crown diameter of 3.13 mm along the horizontal plane and a crown height of 1.65 mm, as measured from CT slices. The teeth in the more labial row are on average larger than those in the more lingual row, although the difference is minor when excluding the antepenultimate tooth of the labial row. The anterior part of the dentary, approximately corresponding to the premaxilla in the upper jaw, is edentulous.

The right maxilla has 12 teeth in two rows, and a tooth position belonging to the third row. Seven of the teeth are in the labial row and 5 in the middle row. The third-row tooth position is located lingual to the middle row and houses a partly formed tooth crown that is much smaller than the space and tilted about 90 degrees relative to other tooth crowns. The hidden left maxilla seems to have at least 7 and 3 teeth, respectively, in the labial and middle rows, and again there seems to be a single tooth in the lingual row.

The tooth crowns are generally rounded, with the crown apex shape ranging from weakly pointed to completely flattened (Figs. 2 and 4c–e), from anterior to posterior and from lingual to labial within the dentition. Most tooth crowns have disto-mesially elongated cross-sectional shapes, except for a few posterior teeth along the jaw margin that are as wide as long in cross-section. All tooth crowns are swollen at least to some extent (Fig. 4c–e), unlike in more derived ichthyosauriforms where molariform teeth are exclusively found in heterodont dentitions with conical anterior and molariform posterior teeth. Cross-sectional images also reveal that the tooth crowns are unusually thin-walled, as judged by the distribution of dense material that at least contains the enamel (Fig. 4a–e). However, the CT images do not allow clear separation of the dentine from the enamel and the rock matrix, which may have high density depending on the place probably because of concretion. Also, the gray levels in the images seem to be driven by artifacts, such as beam hardening and scattering than the actual density of the material, especially near the boundary of bones and teeth. For example, inside the dense wall, the teeth in Fig. 4c–e are least dense (i.e., dark) near the crown apex and becomes denser toward the bottom but this change unlikely to reflect biological structures. Also, the distributions of the darkest parts are inconsistent across images (compare Fig. 4c with e). Thus, the “wall” in the current context means the periphery of the teeth with high density. The root is present in most teeth. Many of the teeth are constricted between the root and crown, even in the largest teeth in the postero-labial region (yellow arrows in Fig. 4). The root is approximately cylindrical without any fluting or infoldings that are typically found in ichthyopterygians.

The dentigerous region is 18.9 mm long in the right dentary and 14.3 mm in the right maxilla. Thus, some dentary teeth have no corresponding teeth in the upper jaw. These teeth are found in the most anterior part of the dentary dentition, where the gap between the right and left mandibular rami is narrow. See Discussion.

### Relative tooth size and crown shape index

Massare (1987) proposed a combination of two tooth characteristics to divide the feeding guilds of Jurassic and Cretaceous marine reptiles, namely the crown shape index quantified as the ratio between the height and diameter of the largest tooth crown in the dentition, and the relative tooth size, measured as the maximum dimension of the largest tooth crown to the skull width^12^. These two metrics were estimated for the present specimen.

The estimation of the tooth size index involves the skull width, which is ideally measured between the quadrates but may be substituted by the width between the lateral sides of the supratemporals in ichthyosauriforms, where the two values are usually similar to each other. Unfortunately, one each of the quadrate and supratemporal has been displaced, making direct measurements of the skull width unreliable. However, a half of the skull width can be measured on the right side of the skull roof that retains its articulation, providing a value of 15.1 mm between the lateral margin of the supratemporal and the sagittal line, measured perpendicular to the latter. This results in an estimated skull width of 30.2 mm.

The crown shape index based on the largest dentary tooth is 1.90, placing *Cartorhynchus* in the crushing guild characterized by an index value above 1.0. The relative tooth size is about 0.104, again placing *Cartorhynchus* in the crushing guild, characterized by values equal to or greater than 0.1.

### Jaw symphysis

The tooth orientation and size, together with the morphology of the anterior tip of the dentary, make it difficult for the right and left mandibular rami to form a solid symphysis. The teeth occupy much of the medial surface of the mandible anteriorly, leaving only the very tip of the snout for possible symphyseal articulation. Yet, the rostral tip of the mandible is slender and curved upward, without a symphyseal surface on the medial side. Therefore, the jaw symphysis, if any, was weak. The jaw symphysis is also weak in Hupehsuchia^13^, the sister taxon of Ichthyosauriformes, so the weakness in *C. lenticarpus* is phylogenetically reasonable.

### Cranial morphology

The CT imaging illuminated misidentifications of bone sutures and one misinterpretation of morphology in the original description^8^. The parietal seems to have the parietal ridge and slope^14^ based on the CT images. The premaxilla has a short supranasal process that forms the anterior-dorsal margin of the external naris. Also, the right quadrate, which was thought to underlie the right quadratojugal, seems to have been dislocated, and most of what was identified as the quadrate is parts of the quadratojugal and squamosal.

### Ancestral state reconstruction

Ancestral state reconstruction based on likelihood suggests that common ancestors along the main lineage of Ichthyosauriformes likely retained conical teeth, and that rounded teeth most likely evolved five times in ichthyosauriforms (Fig. 1a). The parsimony reconstruction suggests that molariform teeth evolved three to five times in ichthyosauriforms, and that the common ancestors along the main lineage of Ichthyopterygia unambiguously retained conical teeth (Fig. 1b).

## Discussion

The new data on the dentition and jaw design in *Cartorhynchus lenticarpus* provides useful information on the feeding ecology of the species. The presence of rounded teeth implies durophagy, although this does not mean exclusive feeding on hard-shelled invertebrates; durophagous marine reptiles and mammals consume various mixtures of hard and soft prey items^15^. Some mixture of hard and soft prey is therefore likely for *C. lenticarpus* too. Such a diet does not contradict the suction-feeding habit that was previously inferred for *C. lenticarpus* based on the co-existence of a robust ceratohyal and short and narrow snout with edentulous tips^8^. For example, among extant marine vertebrates, at least some sparid fishes are known to suction feed despite their molariform teeth and durophagous diet^16^. The prey size of *C. lenticarpus* is inferred to have been small, given that the edentulous tip of the snout, which is likely used as the opening during suction, is about 6 mm wide. The jaw width at the point where the first tooth becomes visible in Fig. 3a is about 10 mm. The narrowness relative to the skull width further supports the inference of suction-feeding because it allows a high degree of pressure concentration along the skull, as seen in extant suction-feeders^17^. No stomach content is available but small thin-shelled bivalves, together with bivalved-arthropods (Thylacocephala) and small ammonoids, occur abundantly in the strata that yielded the holotype of the species^18^. Also, the first diversification peak of invertebrates, especially bivalves, after the end-Permian mass extinction was recorded in Chinese strata coinciding with the occurrence of *C. lenticarpus* and other oldest ichthyosauromorphs^19,20^.

The strange tooth orientation and ‘occlusion’ seen in the specimen is enigmatic—the teeth are structurally strongest when stressed from the tip, not from the side, while high stress is expected when durophages crush hard-bodied prey^21^. There are two possibilities that may reconcile tooth morphology and tooth mechanics, although neither can explain the positions of the tooth wears. First, if the mandible had been plastically ‘twisted’ during preservational compaction, the dentigerous part of the dentary may have been more horizontal in life. However, there is no way to test this hypothesis at this point. Second, if the teeth had been supported by hard tissues that oriented them more vertically in life and the tissues were selectively compressed during diagenesis after elasticity was lost due to the disintegration of collagen, tooth orientation would have been altered. This interpretation would require a substantial mass of porous and compressible hard tissues, possibly resembling the bones of attachment, to support the teeth. However, there is currently no direct evidence to support this interpretation.

The presence of anterior dentary teeth without corresponding upper teeth is puzzling, given that these anterior teeth are almost pointing at each other right to left. Considering that the weak jaw symphysis would potentially allow the two mandibular rami to move relative to each other, it may be tempting to contemplate that the right and left dentary teeth were used against each other. However, the teeth show no signs of tooth wear unlike the more posterior teeth that were used against maxillary teeth. Also, the mechanism to allow such jaw movements, especially muscles, is lacking. Therefore, these teeth were probably not used against other teeth.

Among ichthyopterygians, tooth shape and arrangements that are most similar to those of *Cartorhynchus lenticarpus* are found in *Xinminosaurus catactes*^22^, for having multiple tooth rows with rounded crowns posteriorly in both the upper and lower jaws. To date, *X. catactes* is the only ichthyopterygian with multiple tooth rows in the mandible, although it is possible that some other taxa, such as *Grippia*, may also have the feature—the medial side of the dentary is poorly known in many taxa. Despite the presence of multiple tooth rows, there is a clear difference in tooth-size arrangement between *C. lenticarpus* and *X. catactes*: labial teeth are large in the former where the lingual teeth are larger in the latter. Other differences are found in the anterior dentition and the root morphology–unlike *C. lenticarpus*, *X. catactes* has conical anterior teeth that make its dentition heterodont, while the root has a plicate dentine wall.

The dental morphology of *C. lenticarpus* partly resembles that of *Omphalosaurus*^23–25^ to some extent in that the teeth are rounded. However, unlike in *Omphalosaurus*, most teeth have the root and there is only one tooth per tooth position in *C. lenticarpus*—*Omphalosaurus* is known for vertical overlap of teeth without the root. Thin tooth walls would imply mechanical weakness, which is counter-intuitive given that rounded tooth crowns are often, although not always, used for crushing hard prey.

Molariform teeth evolved in many vertebrate clades, especially in aquatic environments with a variety of hard-shelled invertebrate prey but also on land^26–30^. Of these examples, the best modern analogues for the dentition of *Cartorhynchus lenticarpus* are found in teleost fishes of the Family Sparidae, whose skull length and tooth size are comparable to those of *C*. *lenticarpus*. Some sparids are known for their molariform oral teeth that are arranged in multiple rows per jaw ramus^16,31^. As with *C. lenticarpus*, most of the teeth do not protrude beyond the dentigerous margins of the jaw rami and therefore are largely hidden from external view, although the degree of concealment is greater in *C. lenticarpus*. It may be hypothesized that this concealed placement of the teeth reduces disruption of water movement during suction feeding. Despite the similarities, sparids differ from *C. lenticarpus* in a few respects. As in *Xinminosaurus*, lingual teeth are larger than labial ones, making the tips of the crowns of different tooth rows more leveled. Also, sparids may have anterior prehensile teeth unlike in *C*. *lenticarpus*.

Molariform teeth are usually an ontogenetic feature. Juveniles of relevant species have conical or tricuspid teeth that become larger and more rounded with growth, sometimes becoming almost flattened in extreme cases^32–36^. Such ontogenetic morphological changes are usually associated with dietary shifts^37–39^. A few exceptions to this rule have been noted in at least two, and possibly three species of lizards and one species of fish, where larger juveniles already have at least a few molariform teeth^26,32^. However, we are not aware of a case where juveniles have three or more tooth rows of molariform teeth per oral jaw ramus, or where the labial teeth of juveniles have already become as large as more lingual teeth as a result of tooth replacement. In species with three rows of mandibular teeth, tooth rows are added from the lingual side and teeth are replaced with growth, while the teeth are not yet molariform in small juveniles^31,40^. Therefore, it takes time before molariform teeth reach the labial row and become as large as those in more lingual rows. Given the presence of three tooth rows with large teeth in the labial row, and that molariform teeth are an adult feature in most cases, it is most likely that the holotype of *Cartorhynchus lenticarpus* was not of a juvenile, as previously inferred based on skeletal proportions^8^.

Convergent evolution of molariform teeth within a clade is not rare among non-mammalian vertebrates. For example, at least two lineages within monitor lizards (genus *Varanus*) have molariform teeth in adults, namely an African lineage including *V. niloticus* and the Philippine lineage including *V. olivaceus*^41–43^. Durophagy, associated with oral molariform teeth, evolved ten times independently among moray eels^27^ and up to seven times among sparid fishes^44^. Molariform teeth also repeatedly evolved in the pharyngeal jaws of some fish lineages, such as heroine cichlid fishes where it evolved at least six times^28,45^. Our inference that molariform teeth evolved three to five times in Ichthyosauriformes, whose Linnaean rank is undetermined but should be higher than the Superorder Ichthyopterygia that it contains^46^, is therefore not exceptionally high, especially for an aquatic lineage.

The evolution of molariform teeth is most likely correlated with that of durophagy^21,38,47^. More than half of Early and Middle Triassic ichthyosauriform species had at least a few molariform teeth (Fig. 1), so it is likely that hard-shelled prey formed a substantial proportion of the diet of early ichthyosauriforms. However, it is unlikely that all of them fed on the same prey type through time. For example, molariform ichthyosauriforms in the Early Triassic had small teeth as in *Cartorhynchus*, with the maximum diameter of up to about three millimeters, while those in the Middle Triassic included species with the maximum tooth diameter exceeding 1 cm^22,48^. Also, the shape of the occlusal surface of the crown varied from round to ridged in the Middle Triassic^48^, while Early Triassic species had uniformly rounded teeth. Therefore, it seems that later ichthyosauriforms consumed larger and more diverse prey than their predecessors. This matches the known pattern of invertebrate diversification after the EPME^49,50^. It then appears that ichthyosauriforms exploited the changing communities of hard-shelled prey by repeatedly giving rise to new types of durophages in the Early and Middle Triassic, rather than by a single durophagous lineage adapting to the changes. Therefore, it is likely that the rapid taxonomic diversification of ichthyosauriforms after the EPME was at least partly driven by the evolution of hard-shelled prey.

## Methods

### Specimen

The holotype of *Cartorhynchus lenticarpus* was used. It is accessioned at the Anhui Geological Museum under the specimen number AGB 6257. Its field number was MT-II. The first author is in charge of research at the museum and granted permission to study the specimen himself.

### CT scanning

The holotype of *Cartorhynchus lenticarpus* (AGB 6257) was scanned at the Yinghua testing co. LTD, Shanghai, China, using 250 kV micro CT (V | tome|x s). The specimen was scanned with a beam energy of 180 kV and a flux of 150 mA at a detector resolution of 38 μm per pixel. A total of 1200 transmission images were reconstructed in 1588 slices of 1313 × 720 pixels.

### 3D rendering from CT slices

We used a combination of Fiji^51^ (ImageJ 2.0), 3D Slicer^52^ 4.8.1, and Meshlab^53^ 2016.12 for 3D surface reconstruction given in Fig. 2a,b through a method that may be called Sequential Isosurface Trimming. Fiji was used to crop the slices and to apply a weak convolution filter to reduce noises—the slices suffered from streaks of noises even after the best efforts to remove noise by the scanning facility. Slicer was then used to produce 20 isosurfaces at different gray levels. The resulting isosurfaces were converted to polygonal meshes and saved as PLY files, which were then edited in Meshlab to remove noisy areas—noises typically form linear streaks of random lengths that are parallel to X and Z axes of the original CT images and can readily be distinguished from the smooth surfaces of bones. The trimmed surfaces were then examined for redundancy, and when two or more surfaces were redundant, one with the lowest gray level was retained. The remaining trimmed surfaces were merged and faces with low ambient occlusion (i.e., largely concealed from outside) were removed.

This Sequential Isosurface Trimming approach may not be traditional but we used it because our initial trial using a standard approach of CT image segmentation, whereby bone boundaries are subjectively determined on each CT slice to form the basis of 3D reconstruction, turned out to be unfruitful. It was very difficult to segment bones consistently because of a combination of biases, such as inconsistency of gray levels within and between slices caused by the high-density matrix, high level of noises, and severe compaction of the fossil that turned many bones into thin sheets. The resulting 3D images failed to capture many of the small features.

To test whether Sequential Isosurface Trimming led to unintentional elimination of important bone structures, we also volume-rendered the data in ImageVis 3D^54^ 3.1.0, using the 2D transfer function option for comparison. Supplementary Fig. S1 is an example of such a rendered image. After trying different combinations of 2D transfer functions, we concluded that the surface reconstruction did not lack any significant part of the skeleton that is present in the CT image slices. The outcome of Sequential Isosurface Trimming is given in Supplementary Fig. S2.

### 3D arrangement of the mandibular rami

The mandible was reconstructed by combining the 3D mesh of the right mandibular ramus with the mirror image of itself, given that the left ramus is far less complete than the right. The arrangement of the two was made under the following conditions. First, the mandibular width at the jaw joint was kept at the skull width reported in Results. Second, the anterior tips of the two mandibular rami are located as close to the sagittal plane as possible, without diverging laterally—the tips would diverge from the sagittal plane unless the rami are kept in certain postures because of the strong upward curvature of the mandible. Third, the two mandibular rami do not overlap with each other. The jaw rami were allowed to incline relative to the sagittal plane, but it was evident that there was little space for manipulation under these three conditions. For example, if we try to orient the mandible and maxilla so that their respective teeth point at each other tip-to-tip, both the maxilla and mandibular rami become nearly horizontal, and the mandibular width would become twice as large as the skull width estimated above.

### Ancestral character state reconstruction

We used a recently published phylogenetic hypothesis of Ichthyosauromorpha and its associated taxon-character matrix for ancestral state reconstruction^55^. The matrix reflects the dental morphology reported in the present study, as well as the latest findings on basal ichthyosauromorphs. Dental character states for *Sclerocormus parviceps* are considered ambiguous given that the species may have hidden dentition as in *C. lenticarpus*. The ancestral state reconstruction was performed based on the strict consensus tree of the most parsimonious trees^55^ using Mesquite 3.51^56^. Two reconstruction models, squared-change parsimony^57^ and likelihood models, were used. We only examined character 72, which codes the posterior tooth crown shape as (0) conical, (1) rounded, or (2) flat. In parsimony reconstruction, the multistate character was treated as ordered, while the default model of Mesquite was used in likelihood reconstruction. Because the phylogeny is based on parsimony, the exact branch lengths for likelihood reconstruction is unknown. Thus, branch lengths were kept uniform across the tree when calculating likelihood reconstruction.

## Supplementary information

Supplementary Information.

## Data Availability

All data used in this study are available in Supplementary Material or in cited references.

## References

[1] Kelley, N. P. & Pyenson, N. D. Evolutionary innovation and ecology in marine tetrapods from the Triassic to the Anthropocene. *Science (80-.)*. **348**, 3716-1-3716–7 (2015). 10.1126/science.aaa3716 25883362

[2] Vermeij GJ, Motani R (2018). Land to sea transitions in vertebrates: the dynamics of colonization. Paleobiology.

[3] Benton MJ (2013). Exceptional vertebrate biotas from the Triassic of China, and the expansion of marine ecosystems after the Permo-Triassic mass extinction. Earth-Science Rev..

[4] Motani R (2009). The evolution of marine reptiles. Evol. Educ. Outreach.

[5] Motani R (2005). Evolution of fish-shaped reptiles (Reptilia: Ichthyopterygia) in their physical environments and constraints. Annu. Rev. Earth Planet. Sci..

[6] Motani R (1999). Phylogeny of the Ichthyopterygia. J. Vertebr. Paleontol..

[7] Jiang D-Y (2016). A large aberrant stem ichthyosauriform indicating early rise and demise of ichthyosauromorphs in the wake of the end-Permian extinction. Sci. Rep..

[8] Motani R (2015). A basal ichthyosauriform with a short snout from the Lower Triassic of China. Nature.

[9] Mazin J-M (1981). *Grippia longirostris* Wiman, 1929, un Ichthyopterygia primitif du Trias inférieur du Spitsberg. Bull. du Muséum Natl. d’Histoire Nat..

[10] Motani R (1997). Redescription of the dentition of *Grippia longirostris* (ichthyosauria) with a comparison with *Utatsusaurus hataii*. J. Vertebr. Paleontol..

[11] Motani R (1996). Redescription of the dental features of an early triassic ichthyosaur, *Utatsusaurus hataii*. J. Vertebr. Paleontol..

[12] Massare JA (1987). Tooth morphology and prey preference of Mesozoic marine reptiles. J. Vertebr. Paleontol..

[13] Motani R (2015). Lunge feeding in early marine reptiles and fast evolution of marine tetrapod feeding guilds. Sci. Rep..

[14] Motani R (1999). The skull and taxonomy of *Mixosaurus* (Ichthyopterygia). J. Paleontol..

[15] Kelley NP, Motani R (2015). Trophic convergence drives morphological convergence in marine tetrapods. Biol. Lett..

[16] Vandewalle P, Saintin P, Chardon M (1995). Structures and movements of the buccal and pharyngeal jaws in relation to feeding in *Diplodus sargus*. J. Fish Biol..

[17] Motani R (2013). Absence of suction feeding ichthyosaurs and its implications for Triassic mesopelagic paleoecology. PLoS One.

[18] Ji C, Tintori A, Jiang D-Y, Motani R (2017). New species of Thylacocephala (Arthropoda) from the Spathian (Lower Triassic) of Chaohu, Anhui Province of China. Palaontologische Zeitschrift.

[19] Fan J (2020). A high-resolution summary of Cambrian to Early Triassic marine invertebrate biodiversity. Science (80-.)..

[20] Motani R, Jiang D-Y, Tintori A, Ji C, Huang J-D (2017). Pre- versus post-mass extinction divergence of Mesozoic marine reptiles dictated by time-scale dependence of evolutionary rates. Proc. R. Soc. B-Biological Sci..

[21] Crofts SB, Summers AP (2014). How to best smash a snail: the effect of tooth shape on crushing load. J. R. Soc. Interface.

[22] Jiang D-Y (2008). New primitive ichthyosaurian (Reptilia, Diapsida) from the Middle Triassic of Panxian, Guizhou, southwestern China and its position in the Triassic biotic recovery. Prog. Nat. Sci. Int..

[23] Merriam, J. C. Preliminary note on a new marine reptile from the Middle Triassic of Nevada. *Bull. Dep. Geol. Univ. Calif. Plublications* Bulletin o, 71–79 (1906).

[24] Sander PM, Faber C (2003). The Triassic marine reptile Omphalosaurus: Osteology, jaw anatomy, and evidence for ichthyosaurian affinities. J. Vertebr. Paleontol..

[25] Motani R (2000). Is *Omphalosaurus* ichthyopterygian? A phylogenetic perspective. J. Vertebr. Paleontol..

[26] Bemis KE, Bemis WE (2015). Functional and developmental morphology of tooth replacement in the Atlantic Wolffish, *Anarhichas lupus* (Teleostei: Zoarcoidei: Anarhichadidae). Copeia.

[27] Collar DC, Reece JS, Alfaro ME, Wainwright PC, Mehta RS (2014). Imperfect morphological convergence: variable changes in cranial structures underlie transitions to durophagy in moray eels. Am. Nat..

[28] Hulsey CD, Roberts RJ, Lin ASP, Guldberg R, Streelman JT (2008). Convergence in a mechanically complex phenotype: detecting structural adaptations for crushing in cichlid fish. Evolution (N. Y)..

[29] Grubich J (2003). Morphological convergence of pharyngeal jaw structure in durophagous perciform fish. Biol. J. Linn. Soc..

[30] Gidmark NJ, Taylor C, Lopresti E, Brainerd E (2015). Functional morphology of durophagy in black carp, *Mylopharyngodon piceus*. J. Morphol..

[31] Elgendy SAA, Alsafy MAM, Tanekhy M (2016). Morphological characterization of the oral cavity of the gilthead seabream (*Sparus aurata*) with emphasis on the teeth-age adaptation. Microsc. Res. Tech..

[32] Estes R, Williams EE (1984). Ontogenetic variation in the molariform teeth of lizards. J. Vertebr. Paleontol..

[33] Dessem D (1985). Ontogenetic changes in the dentition and diet of *Tupinambis* (Lacertilis: Teiidae). Copeia.

[34] D’Amore DC (2015). Illustrating ontogenetic change in the dentition of the Nile monitor lizard, *Varanus niloticus*: A case study in the application of geometric morphometric methods for the quantification of shape-size heterodonty. J. Anat..

[35] Fernandez L, Motta PJ, Hernandez LP, Motta PJ (1997). Trophic consequences of differential performance: Ontogeny of oral jaw crushing performance in the sheepshead, *Archosargus probatocephalus* (Teleostei, Sparidae). J. Zool..

[36] Hung NM, Ryan TM, Stauffer JR, Madsen H (2015). Does hardness of food affect the development of pharyngeal teeth of the black carp, *Mylopharyngodon piceus* (Pisces: Cyprinidae)?. Biol. Control.

[37] French B, Platell ME, Clarke KR, Potter IC (2012). Ranking of length-class, seasonal and regional effects on dietary compositions of the co-occurring *Pagrus auratus* (Sparidae) and *Pseudocaranx georgianus* (Carangidae). Estuar. Coast. Shelf Sci..

[38] Constantino PJ, Bush MB, Barani A, Lawn BR (2016). On the evolutionary advantage of multi-cusped teeth. J. R. Soc. Interface.

[39] Richardson TJ, Potts WM, Santos CV, Sauer WHH (2011). Ontogenetic dietary shift and morphological correlates for *Diplodus capensis* (Teleostei: Sparidae) in southern Angola. African Zool..

[40] Leblanc, A. R. H. & Reisz, R. R. Patterns of tooth development and replacement in captorhinid reptiles: A comparative approach for understanding the origin of multiple tooth rows. *J. Vertebr. Paleontol*., 10.1080/02724634.2014.919928 (2015).

[41] Berkovitz, B. & Shellis, P. Reptiles 2. In *The Teeth of Non-Mammalian Vertebrates* 201–224. 10.1016/B978-0-12-802850-6.00007-2 (2017).

[42] Pianka, E. R., King, D. & King, R. A. Varanoid lizards of the world. (Indiana University Press, 2004).

[43] Edmund, A. G. Dentition. In Biology of the Reptilia. Volume I. Morphology A. (eds. Gans, C., Bellairs, A. d’A. & Parsons, T. S.) 1, 117–200 (Academic Press, 1969).

[44] Chiba SN, Iwatsuki Y, Yoshino T, Hanzawa N (2009). Comprehensive phylogeny of the family Sparidae (Perciformes: Teleostei) inferred from mitochondrial gene analyses. Genes Genet. Syst..

[45] Hulsey CD, Darrin Hulsey C (2006). Function of a key morphological innovation: Fusion of the cichlid pharyngeal jaw. Proc. R. Soc. B Biol. Sci..

[46] McGowan, C. & Motani, R. Ichthyopterygia. Handbook of Paleoherpetology **8**, (Verlag Dr. Friedrich Pfeil, 2003).

[47] Hanel R, Sturmbauer C (2000). Multiple recurrent evolution of trophic types in northeastern Atlantic and Mediterranean seabreams (Sparidae, Percoidei). J. Mol. Evol..

[48] Motani R (2005). Detailed tooth morphology in a durophagous ichthyosaur captured by 3D laser scanner. J. Vertebr. Paleontol..

[49] Foster WJ, Sebe K (2017). Recovery and diversification of marine communities following the late Permian mass extinction event in the western Palaeotethys. Glob. Planet. Change.

[50] Hofmann R (2014). Recovery of benthic marine communities from the end-Permian mass extinction at the low latitudes of eastern Panthalassa. Palaeontology.

[51] Schindelin J (2012). Fiji: an open-source platform for biological-image analysis. Nat. Methods.

[52] Fedorov A (2012). 3D Slicer as an image computing platform for the Quantitative Imaging Network. Magn. Reson. Imaging.

[53] Cignoni, P. *et al*. MeshLab: an open-source mesh processing tool. *Sixth Eurographics Ital. Chapter Conf*., 10.2312/LocalChapterEvents/ItalChap/ItalianChapConf2008/129-136 (2008).

[54] Fogal, T. & Krüger, J. Tuvok, an Architecture for Large Scale Volume Rendering. In Proceedings of the 15th International Workshop on Vision, Modeling, and Visualization (2010).

[55] Huang J-D (2019). The new ichthyosauriform *Chaohusaurus brevifemoralis* (Reptilia, Ichthyosauromorpha) from Majiashan, Chaohu, Anhui Province, China. PeerJ.

[56] Maddison, W. P. & Maddison, D. R. Mesquite: a modular system for evolutionary analysis. Version 3.2. http://mesquiteproject.org (2017).

[57] Maddison WP (1991). Squared-change parsimony reconstructions of ancestral states for continuous-valued characters on a phylogenetic tree. Syst. Biol..

