# Supplementary material for: Repeated evolution of durophagy during ichthyosaur radiation after mass extinction indicated by hidden dentition
Source: Sci Rep. 2020 May 8;10:7798. doi: 10.1038/s41598-020-64854-z (PMC7210957; doi:10.1038/s41598-020-64854-z)
Supplement: Supplementary file 1 — Supplementary Information. [file 41598_2020_64854_MOESM1_ESM.docx]

# Supplementary files for:

“Repeated evolution of durophagy during ichthyosaur radiation after mass extinction indicated by hidden dentition”

Jian-dong Huang^1^, Ryosuke Motani^2,*^, Da-yong Jiang^3^, Xin-xin Ren^4^, Andrea Tintori^5^, Olivier Rieppel^6^, Min Zhou^3^, Yun-chao Hu^1^, and Rong Zhang^1^

^1^Department of Research, Anhui Geological Museum, Jiahe Road 999, Hefei, Anhui, 230031 China.

# Table of Contents

- Supplementary Fig. S1. (p. 2)
- Supplementary Fig. S2 (p. 3)


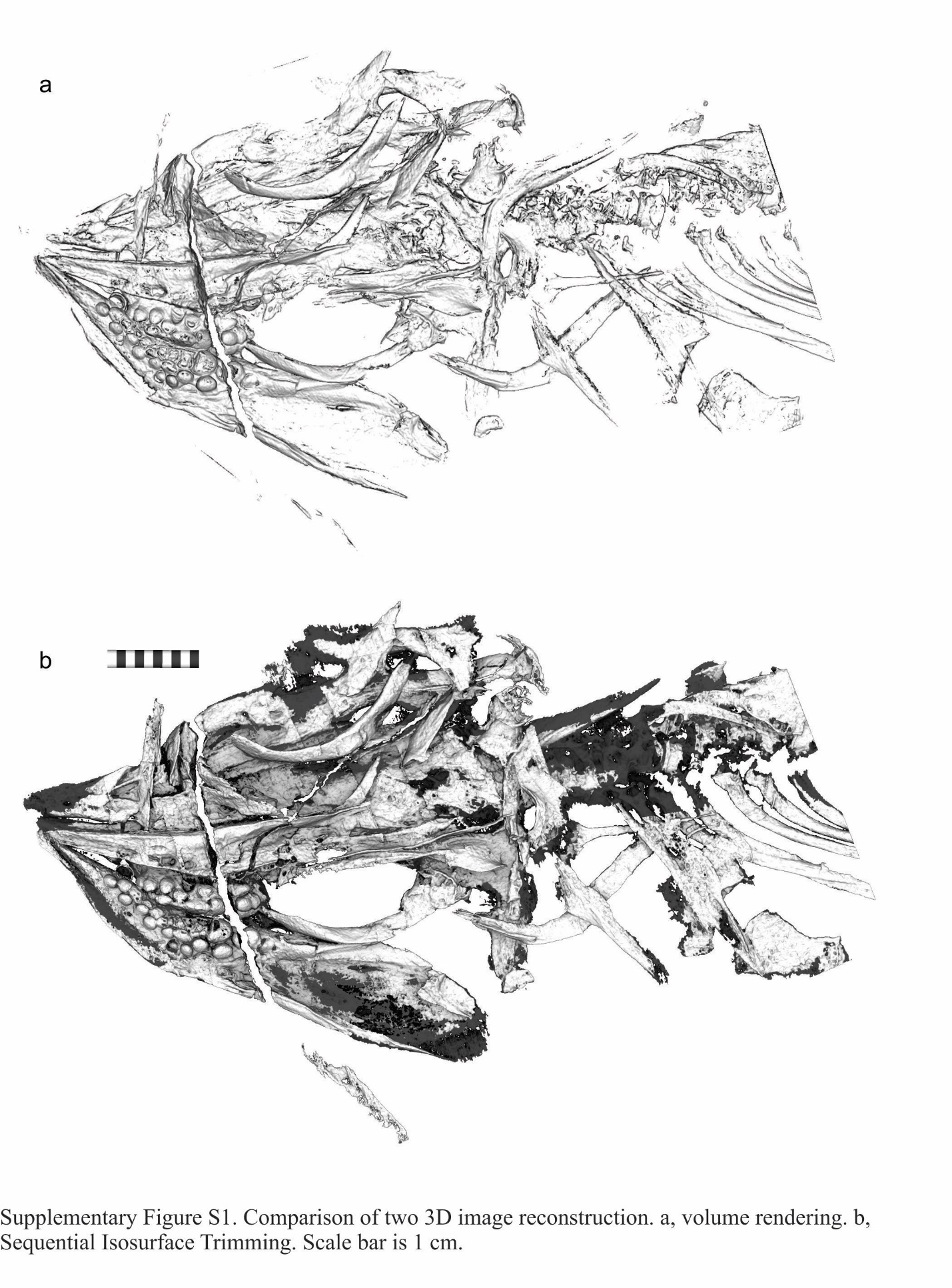


**
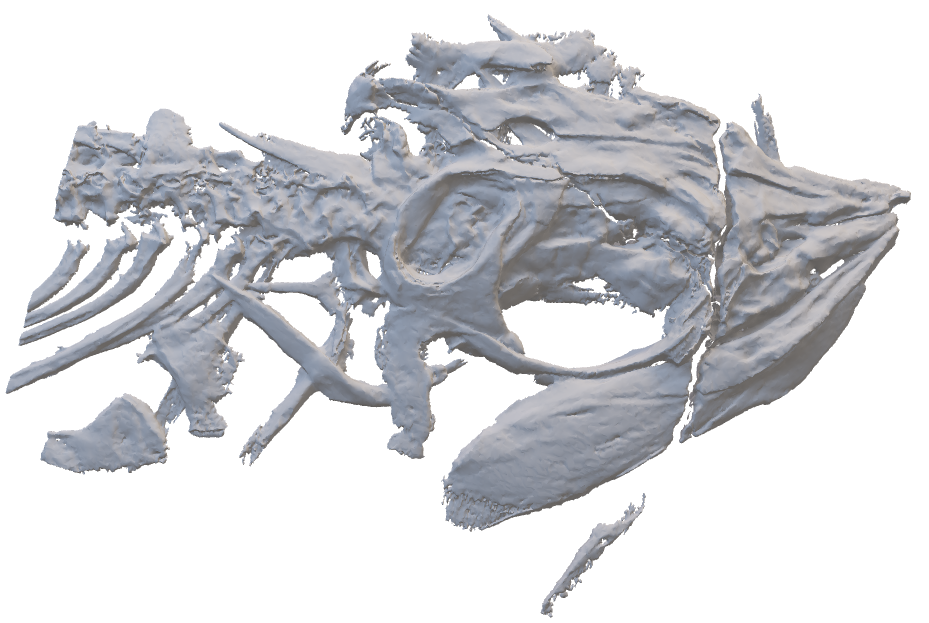
**

**Supplementary Figure S2**. 3D model resulting from Successive Isosurface Trimming. It has been decimated to about 6.25% of the original resolution to achieve a sufficiently small file size.

**
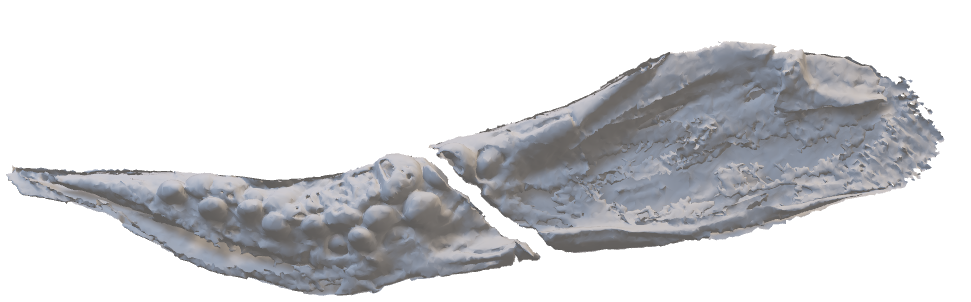
**

**Supplementary Figure S2**. 3D model of the mandible. It has been decimated to about 6.25% of the original resolution to achieve a sufficiently small file size.
